# Idiopathic thrombocytopenic purpura in women with breast cancer

**Published:** 2010

**Authors:** Mozhgan Alam Samimi, Nooshin Mirkheshti, Mitra Heidarpour, Mozhgan Abdollahi

**Affiliations:** a Assistant Professor of Hematology, Sayyed Al-Shohada Hospital, Isfahan University of Medical Sciences, Isfahan, Iran; b Research Assistant, East Sage Investigative Corporation, Isfahan Science and Technology Town, Isfahan, Iran; c Assistant Professor of Pathology, Pathology Department, Al-Zahra Hospital, Isfahan University of Medical Sciences, Isfahan, Iran; d General Practitioner, Sayyed Al-Shohada Hospital, Isfahan University of Medical Sciences, Isfahan, Iran

**Keywords:** Idiopathic Thrombocytopenic Purpura, Breast Cancer

## Abstract

The association of solid tumors with idiopathic thrombocytopenic purpura (ITP) is rare. However, there have been some case reports indicating an association between breast cancer and ITP. In this article four patients with breast cancer and ITP are mentioned. The diagnosis of breast cancer was based on the results of biopsy or surgical sample. The ITP diagnosis criteria were 1) exclusion of drug induced thrombocytopenia, 2) platelet count less than 140 × 109 /l with normal or increased number of megakaryocytes on bone marrow samples, and 3) absence of splenomegaly. In this case report an association of breast cancer and ITP is shown.

Thrombocytopenia is one of the disorders that has been observed in different cancers and some mechanisms has been supposed as a reason of this disorder including tumor bone marrow infiltration, chemotherapy/radiotherapy induced hypoplasia, disseminated intravascular coagulation^1^ or thrombotic thrombocytopenic purpura (TTP).^2^

TTP^3^ and ITP^4^,^5^ have been described in patients with malignant diseases. For example, the association between ITP and lymphoid neoplasms, has been observed in some studies.^6^,^7^ However, regardless lymphoid neoplasms, concomitant solid tumors and ITP have been reported in a limited number of studies. Most of these observations are relevant in breast cancer. Searching to find a relationship between ITP and breast cancer has been raised in recent years,^8^ because such relationship can be considered as a predictive or prognostic value in breast cancer.

In this article, 4 cases of patients with breast cancer and ITP treated in the Department of Medical Oncology of the Sayyed Al- Shohada hospital are reported. Clinical and laboratory characteristics of the two diseases have been reported in this article.

## Case Report

All patients registered to treat for breast cancer at the Sayyed Al- Shohada hospital between 2005 and 2007. The diagnosis of breast cancer was confirmed by biopsy or surgical sample. Tumor size, axillary lymph node involvement, histologic type and hormonal receptor status were determined for all patients. Clinical examination, blood count, liver function tests, chest radiograph, mammogram, liver ultrasound or computed tomography studies, and bone marrow biopsy were conducted for each patient. According to the study done by Peffault et al, 8 the ITP diagnosis criteria were: 1) exclusion of drug induced thrombocytopenia, 2) platelet count less than 140 × 10^9^ /l with normal or increased number of megakaryocytes on bone marrow samples, and 3) absence of splenomegaly. The antiplatelet antibody was not considered to be an absolute diagnostic criterion.^8^,^9^

In all patients, there was no history of prior blood transfusions, recent medications, or human immunodeficiency virus risk factors. Pertinent negative findings on physical examination included no evidence of lymphadenopa- thy, splenomegaly, ecchymoses, or petechiae.

### 

#### Case 1

**Figure 1:**
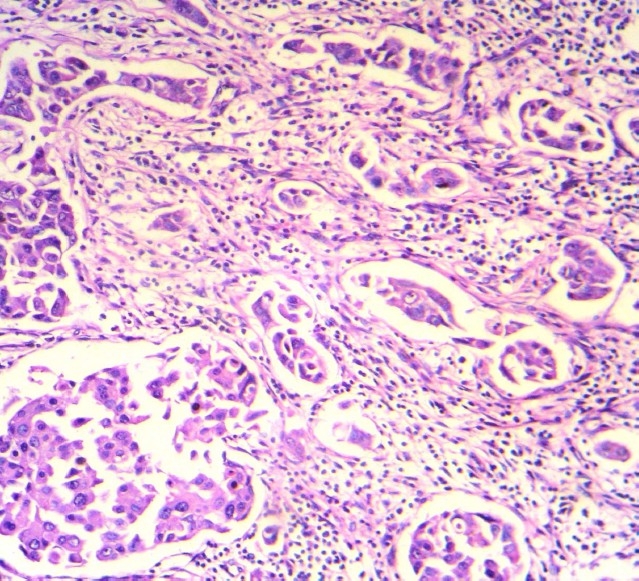
Histological findings of the surgical specimen of the left breast tumor show invasive ductal carcinoma (hematoxylin-eosin stain, 100 ×)

A 73 year- old woman presented with a lump in her left breast underneath the nipple. An excisional biopsy revealed invasive ductal carcinoma grade III (Figure 1). Estrogen and progesterone receptors were positive.

At presentation complete blood count showed a marked thrombocytopenia without any evidence of a leukoerythroblastic reaction; the white blood cell (WBC) count and hemoglobin level were normal (hemoglobin: 13 g/dl, WBC: 5 × 10^9^ /l, platelet count: 70 × 10^9^ /l). A prior complete blood count showed a hemoglobin level of 14 g/dl, WBC 55 × 10^9^ /l, and platelet count of 200 × 10^9^ /l.

Laboratory workup included a negative anti- nuclear antibody, and no evidence of disseminated intravascular coagulopathy. Bone marrow biopsy was normal, with a normal number of megakaryocytes; no dysplastic changes were noted.

Adjuvant chemotherapy of this patient included: 4 courses of cyclophosphamide and adriamycin and 4 courses of taxol. A clinical diagnosis of idiopathic thrombocytopenic purpura (ITP) was made after six months of last chemotherapy administration. No therapy began regarding the platelet count greater than 50 × 10^9^ /l. In 3- year follow up, the platelet count remained at the same level. There was no evidence of recurrence of cancer.

#### Case 2

A 54 year- old woman presented with a lump in her right breast underneath the nipple. An excisional biopsy revealed inflammatory lobular carcinoma. Estrogen receptors were negative and progesterone receptors were positive.

Twelve months after the chemotherapy for relapse, complete blood count showed a mild to moderate thrombocytopenia without any evidence of a leukoerythroblastic reaction; the white blood cell (WBC) count and hemoglobin level were normal and platelet count was 90 × 10^9^ /l. A prior complete blood count showed a platelet count of 170 × 10^9^ /l.

The adjuvant chemotherapy used in this patient included: 4 courses of cyclophosphamide and adriamycin and 4 courses of taxol. It must be mentioned that there was no history of leukoerythroblastic reaction by previous chemotherapy. The chemotherapy regiment was the same in both times (the primary tumor and in its relapse).

Laboratory workup included a negative anti- nuclear antibody, and no evidence of disseminated intravascular coagulopathy. The patient had a positive serum platelet antibody reacting against glycoprotein IIb/IIIa. Bone marrow biopsy was normal, with a normal number of megakaryocytes; no dysplastic changes were noted.

A clinical diagnosis of idiopathic thrombocytopenic purpura (ITP) was made. No therapy began regarding the platelet count greater than 50 × 10^9^ /l. In 3- year follow up, the platelete count remained at the same level.

#### Case 3

A 41 year- old woman presented with a lump in the lateral upper quadrant of her left breast. An excisional biopsy revealed in situ papillary carcinoma. Estrogen receptor was negative and progesterone receptor were positive.

A complete blood count while she was on tamoxifen for three years showed thrombocytopenia without any evidence of a leukoerythroblastic reaction; the white blood cell (WBC) count and hemoglobin level were normal (hemoglobin of 12.5 g/dl, WBC 6 × 10^9^ /l, and platelet count of 54 × 10^9^ /l). A prior complete blood count showed a hemoglobin level of 13 g/dl, WBC 5 × 10^9^ /l, and platelet count of 300 × 10^9^ /l. There was no history of prior blood transfusions, and recent medications other than tamoxifen.

Laboratory workup included a negative anti- nuclear antibody, and no evidence of disseminated intravascular coagulopathy. The patient had a positive serum platelet antibody reacting against glycoprotein IIb/IIIa. Bone marrow biopsy was normal, with a normal number of megakaryocytes; no dysplastic changes were noted.

A clinical diagnosis of idiopathic thrombo- cytopenic purpura (ITP) was made. No therapy began regarding the platelet count greater than 50 × 10^9^ /l. In 3- year follow up, the platelete count remained at the same level.

#### Case 4

**Figure 2:**
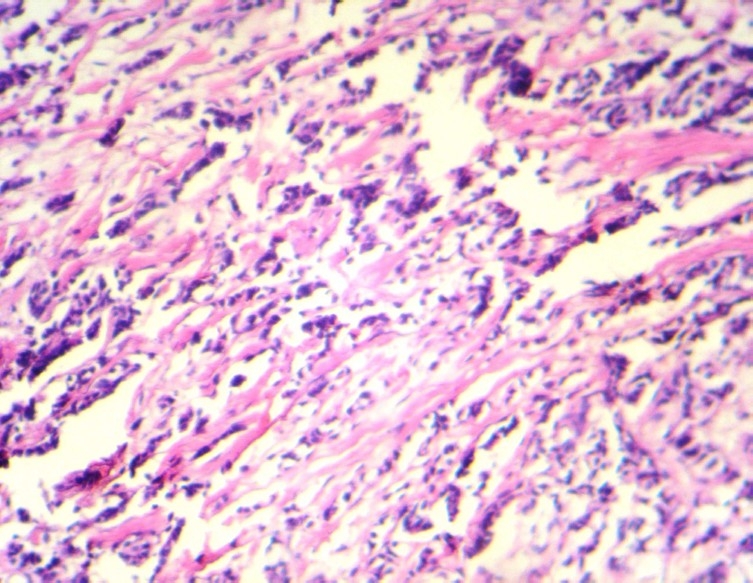
Histological findings of the surgical specimen of the left breast tumor show invasive lobular carcinoma (hematoxylin-eosin stain, 100 ×)

A 40 year- old woman presented with a lump in her left breast underneath the nipple. An excisional biopsy revealed invasive lobular carcinoma (Figure 2). Estrogen and progesterone receptors were positive.

Three months after the end of TAC (taxotere and adriamycine and cyclophosphamide) regiment chemotherapy complete blood count showed a marked thrombocytopenia without any evidence of a leukoerythroblastic reaction; the white blood cell (WBC) count and hemoglobin level were normal (hemoglobin of 13 g/dl, WBC of 5 × 10^9^ /l, and platelet count of 25 × 10^9^ /l). A prior complete blood count showed a hemoglobin level of 13.5 g/dl, WBC 7 × 10^9^ /l, and platelet count of 150 × 10^9^ /l.

Laboratory workup included a negative anti- nuclear antibody, and no evidence of disseminated intravascular coagulopathy. The patient had a positive serum platelet antibody reacting against glycoprotein IIb/IIIa. Bone marrow biopsy was normal, with a normal number of megakaryocytes; no dysplastic changes were noted.

A clinical diagnosis of idiopathic thrombocytopenic purpura (ITP) was made. A regimen of 60 mg of prednisone daily was begun. Follow- up complete blood counts 2 weeks later showed platelet count of 160 × 10^9^ /l. Prednisolone continued and tapered after about 3 months. Platelet count became normal after using prednisolone.

## Discussion

In this article, 4 cases with ITP and breast cancer that were diagnosed and treated at the Sayyed Al- Shohada hospital were described. While ITP is still one of the most common autoimmune disorders, its relationship with solid tumors has not certainly been confirmed by conducted researches. Similarly, ITP has been reported in a small number of patients with breast cancers^4^,^10^–^14^ and the study done by Peffault de Latour et al in 2004 is the largest series reported in the literature, that includes 10 patients with ITP and breast cancer.^8^ In the current study 4 patients with 4 different types of breast cancer and positive hormonal receptor status were presented. Considering this and all previous publications, positive hormonal receptor status (estrogen and/or progesterone receptors), especially progester one receptors, appeared to be more frequently associated with ITP. Despite some other publications, the current patients did not present a long time interval between the two diseases.^8^ Few reports have suggested that thrombocytopenia occurs in the case of breast cancer progression and particularly bone metastases^10^,^12^; but in the present study, no poor outcome was observed when breast cancer is associated with ITP. So, more aggressive therapeutic strategy for these patients that is suggested in some articles does not seem necessary.

The relationship between ITP and breast cancer raises the question concerning the mechanisms involved in this association. In solid tumors, thrombocytopenia is known to be caused by bone marrow hypoplasia secondary to chemotherapy, bone marrow replacement by tumor, or platelet destruction caused by tumor- intrinsic factors activating the coagulation cascade.^12^ Horváth et al reported the presence of circulating immune complex in 62% patients with breast cancer.^15^ In addition, it is mentioned in some studies, all cases with ITP and breast cancer had stage IV breast cancer and this might suggest that a high tumor burden is required to produce an immune response resulting in platelet destruction. But regarding the lower stages of breast cancer that is reported in some other studies, like the present one, this mechanism can’t be certainly involved.

The underlying mechanism for the development of ITP in breast cancer is unclear and further studies are needed to understand this exceptional but relevant association.
